# Vitamin D and Its Role as a Protective Factor in Allergy

**DOI:** 10.1155/2014/951946

**Published:** 2014-08-31

**Authors:** Mehmet Hoxha, Maria Zoto, Leonard Deda, Gentian Vyshka

**Affiliations:** ^1^Service of Allergology and Clinical Immunology, UHC “Mother Teresa,” Tirana, Albania; ^2^Biomedical and Experimental Department, Faculty of Medicine, University of Medicine in Tirana, Tirana 1005, Albania

## Abstract

The relationship between vitamin D status and asthma has been subject to several studies in the last decade. Epidemiological data suggest that incidence of asthma and atopic diseases increased significantly in most Westernized countries. The significant variation between countries suggests that besides genetic factors, environmental aspects play a role in the pathogenesis of atopy. The prevalence of hypovitaminosis D is high in many industrialized countries. In addition to its relationship with bone metabolism, vitamin D is recognized as an immunomodulator, with important effects on both adaptive and innate immunity. Correlations between vitamin D status and asthma have been formulated, with a considerable interest in assessing whether this vitamin protects against or reduces asthma morbidity. In this review, we discuss recent findings regarding vitamin D status throughout Europe and its influence over asthma and allergic rhinitis prevalence. Geographical latitude and dietary habits may explain the lower prevalence of allergic disease in Albania. We also consider the effects of vitamin D supplementation in allergic disease. Several clinical trials are under way and their results are needed in order to make definitive recommendations about the optimal dose of vitamin D for prevention and treatment of asthma and allergic disease.

## 1. Introduction

Vitamin D is a hormone with multiple physiological actions, many effects of which have been found to occur outside its classical role in calcium homeostasis. We now know that vitamin D receptors (VDRs) are expressed in many cell types [1], including various immune cells, suggesting the role of vitamin D on immune system [2]. These recent findings have increased interest in vitamin D status and its link to several nonskeletal diseases [1].

**Figure 1:**
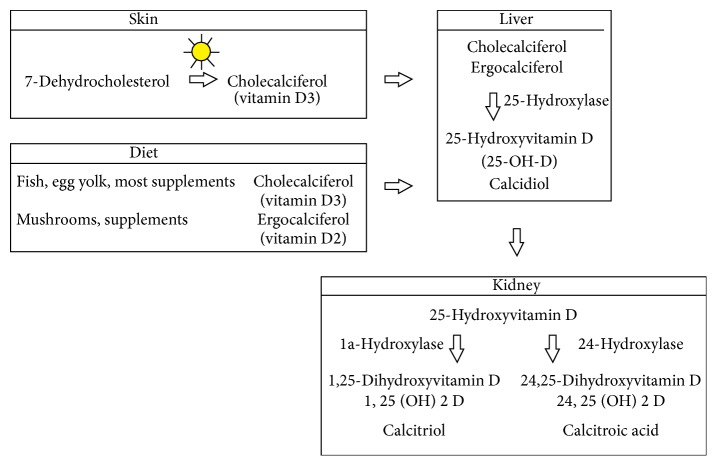
Metabolism of Vitamin D.

The main source of vitamin D in humans is solar UV-B (290–315 nm wavelengths) radiation, which influences the formation of previtamin D in the skin (cholecalciferol). Cholecalciferol from the skin or derived from nutrition is metabolized in the liver to 25-hydroxyvitamin D (25-OH-D). This is the major circulating form; thus it is usually used to measure serum vitamin D levels. 25-OH-D is then transported to the kidneys where it is metabolized to its active form calcitriol (1,25(OH)D,1,25-dihydroxyvitamin D) (Figure 1) [2, 3]. Although thresholds of serum 25(OH)D are still debated, guidelines from the Institute of Medicine (IOM) for bone health define “vitamin D deficiency” as serum 25(OH)D levels below 30 nmol/L (<12 ng/mL), while sufficient vitamin D levels should be considered serum levels of at least 50 nmol/L (20 ng/mL) [4]. Due to evidence of vitamin D insufficiency on allergic disease prevalence, many researchers categorized vitamin D sufficiency as >75 nmol/L (30 ng/mL) [1].

Since 1999, when Wjst and Dold were the first scientists to hypothesize a link between vitamin D and allergic diseases, two conflicting hypotheses are raised. The first hypothesis tries to correlate high serum vitamin D levels with the rise in prevalence of allergies and asthma [5]. The authors suggest that the geographic trend of higher disease prevalence in more developed countries runs in parallel with vitamin D exposure [5]. The first study that investigated this possible connection was a birth cohort study conducted in Finland. The authors found that higher risk for atopy, allergic rhinitis, and asthma was associated with increase in vitamin D supplementation for newborns in order to prevent infantile rickets [6]. A second hypothesis developed later suggested that vitamin D deficiency may contribute to the recent increase in allergies in Western countries [7, 8]. There is an increasing body of evidence to support the hypothesis that this widespread vitamin D deficiency correlates with atopy, asthma, and food allergy [8–12]. Prevalence of hypovitaminosis D varies among different countries and among different population groups within a given country and for each population over time. In many industrialized countries, up to 50% of the population has insufficient vitamin D [1]. Based on one study, vitamin D insufficiency in the United States increased from 55% to 77% between data collection ranges of 1988–1994 to 2001–2004 [13].

There is a combination of different factors which determine 25(OH)D serum levels and vitamin D deficiency like skin pigmentation, low sun exposure, more time spent indoors, obesity, higher latitudes, and winter season [13, 14]. Other secondary causes that could affect vitamin D serum levels are diseases including rheumatoid arthritis, cystic fibrosis, ulcerative colitis, Crohn's disease, celiac disease, rickets, and medications [15].

In this review, we outline the basic metabolism of vitamin D and its effects on the immune system. In addition, we discuss recent findings regarding vitamin D status and its relation to allergy, specifically throughout Europe and Mediterranean countries. We also considered the effects of vitamin D supplementation in allergic disease, highlighting the recent recommendations.

## 2. The Effects of Vitamin D on the Immune System

The human immune system is divided into two branches: adaptive and innate immunity. There is plenty of evidence to show that vitamin D has significant effects on both of them. Its immunomodulatory role has been recognized recently with the discovery of vitamin D receptor (VDR) and the hydroxylation of 25(OH)D on distinct cell types. VDRs have been identified in many tissues and cells in the human body, including nearly all cells of the immune system (T cells, B cells, neutrophils, macrophages, and dendritic cells) [16]. It has been demonstrated that vitamin D affects several aspects of innate immunity. Vitamin D inhibits the expression of TLR (Toll-like receptor) on monocytes, inhibits proinflammatory cytokine production, and induces antimicrobial peptide synthesis [17, 18]. Vitamin D also impacts the adaptive immune system, specially affecting T-cell activation and antigen-presenting cells function. In recent studies, vitamin D is associated with reduction of Th1 cytokine secretion and inhibition of T cells proliferation. The association of vitamin D and Th2 cells is less clear and contradictory, with report of both increased and decreased expression of the Th-2 cytokines IL-4, IL-5, and IL-10 in adult peripheral blood cell cultures [19, 20]. In conclusion, it seems that vitamin D has a key role in Th1-Th2 balance, which could be relevant in allergic disease.

## 3. Vitamin D, Asthma, and Allergic Rhinitis

Vitamin D deficiency has been blamed as a cause of increased incidence of asthma and allergy symptoms. In a study conducted by Hollams et al. in Australia, 689 subjects were seen longitudinally at both ages of 6 and 14 years [21]. This study showed that vitamin D levels at ages 6 and 14 years were predictive of allergy/asthma outcomes at both ages, but more importantly, vitamin D levels at age 6 years were predictive of subsequent atopy and asthma-associated phenotypes at age 14 years. This is the first study which demonstrates the association between vitamin D and asthma in older children, comparing with the early-life birth cohort studies.

In addition to the relationship between vitamin D status and asthma, there is considerable interest in assessing whether this vitamin protects against or reduces asthma morbidity. It is now well known that there is a significant association between vitamin D deficiency and infections. This association becomes particularly significant in children with respiratory disease such as asthma. The most common causes of acute asthma exacerbations are viral upper respiratory tract infections. The human rhinovirus (HRV) is the commonest trigger for acute asthma. Up to 80% of asthma exacerbations are triggered by a “cold.” A recent clinical trial showed that vitamin D supplementation (500 IU/day) given as adjuvant therapy to inhaled corticosteroids in children with asthma reduced the risk of asthma exacerbation triggered by respiratory tract infections [22]. Other researchers in Costa Rica studied vitamin D levels in children with asthma and demonstrated that lower vitamin D levels were associated with increased airway responsiveness, higher eosinophilic counts and total IgE levels, and increased risk of severe asthma exacerbations [23]. This finding suggests that sufficient vitamin D levels may help to control infections and reduce inflammatory responses, resulting in viral infections causing less severe symptoms. The same authors conducted a longitudinal study based on Childhood Asthma Management Program and showed that the group with the lower risk of exacerbations was the group with 25(OH)D ≥ 30 ng/mL and who were receiving inhaled corticosteroids [24]. The hypothesis that vitamin D supplementation might potentiate the anti-inflammatory function of corticosteroids is intriguing because glucocorticoid resistance is an important obstacle to effective treatment in some patients with asthma. Searing et al. in their study of asthmatic children demonstrated a significant association between lower vitamin D levels and greater use of inhaled or oral corticosteroids and total steroid dose [25]. Similar results were obtained in studies conducted on asthmatic adults [26]. Xystrakis et al. demonstrated the same association in vitro by using cell cultures obtained from steroid sensitive and steroid-resistant asthmatic subjects. Adding vitamin D to CD4+ T-cell cultures from steroid-resistant patients enhances the response to dexamethasone by inducing the production of IL-10 [27]. Furthermore, they showed that oral administration of vitamin D in severe asthmatics inverted steroid resistance through induction of IL-10-secreting Tregs (regulatory T-cells). These observations, together with clinical and experimental studies, justify the use of vitamin D in the treatment of severe asthma, particularly to enhance action of steroids.

Another aspect involved in the relationship between vitamin D deficiency and asthma relates to lung function impairment. Consistent with the role of vitamin D on enhancing steroid responsiveness, several studies of children and adults have shown that a low vitamin D level is associated with impaired lung function. Children with insufficient vitamin D levels were found to have a slightly lower mean FEV1 than children with sufficient vitamin levels [25]. Other studies in adults show a strong relationship between serum concentrations of vitamin D, forced expiratory volume in 1 second (FEV1), and forced vital capacity, where decreasing pulmonary function is associated with vitamin D deficiency [26, 28].

It has been found that different gene polymorphisms of the vitamin D receptor (VDR) and vitamin D binding protein (VDBP) have variable associations with asthma. Together with different serum levels of vitamin D, also VDR and VDBP variants seem to represent a risk factor for asthma [29]. The vitamin D receptor is present in bronchial smooth muscle cells which are associated with active protein synthesis. It has been demonstrated that vitamin D inhibits bronchial smooth muscle proliferation induced by platelet-derived growth factor and it also influences the microarray gene expression signature in bronchial smooth muscle cells [30–32]. This finding suggests a role of vitamin D in cell growth and survival and morphogenesis and airway remodeling, which may be important in asthma pathophysiology and treatment [32].

Of the different allergic disorders, perhaps asthma has been the most closely examined in the context of vitamin D. Although the underlying mechanisms of how vitamin D modulates the pathogenesis of asthma have not been completely understood, the available data suggest an association between vitamin D deficiency and asthma. On the other hand, there is insufficient and weak evidence for an association between vitamin D status and atopic disease other than asthma. In a cross-sectional study, Hyppönen et al. showed a U-shaped relation between serum vitamin D and total IgE in adults, at 45 years of age. Thus, IgE concentrations were higher for participants with low (<25 nmol/L) and with very high vitamin D serum levels (>135 nmol/L) compared with a reference group (100–125 nmol/L) [33]. Correcting serum concentrations of 25(OH)D to physiological levels reduced the IgE level significantly, further supporting an allergy-protective role for vitamin D in adults. Following patients with chronic rhinosinusitis (CRS), current clinical studies have shown that CRS patients had serum vitamin D levels 40–50% lower than the serum levels in the control group [34, 35]. In a study performed in Iran, vitamin D levels were assessed in 50 patients with allergic rhinitis and the study results were compared with vitamin D status in normal population. The prevalence of severe vitamin D deficiency was higher in patients with allergic rhinitis than in normal population, 30% and 5.1%, respectively [36].

The relationship between vitamin D status and asthma has been the subject of several studies in the last decade. As mentioned in the introduction the prevalence of hypovitaminosis D is high in many industrialized countries. Furthermore, epidemiological studies suggest that atopic diseases increased significantly in most Westernized countries. According to ISAAC Phase Three (1999–2004) prevalence of asthma symptoms in children aged 6-7 years and 13-14 years was, respectively, from <5% to 14.5% and <5% to 11.2% for the east Europe and for west Europe 5.4%–20.9% and 4.1%–27.8%, respectively. On the other hand, the prevalence of allergic rhinoconjunctivitis symptoms in children aged 6-7 years and 13-14 years was <5%–7.1% and ≈5%–19.3% for each group, respectively, at east Europe and 6.2%–11.1% and 7.1%–22.2% at west Europe. In addition, a similar north-south gradient has been observed in Europe for atopic diseases, with countries like Albania and Greece presenting the lowest prevalence [7]. This remarkable variation suggests that environmental factors play an important role in the pathogenesis of allergic diseases. Factors like geographical latitude and Mediterranean diet with fresh fruits, vegetables, and nuts are protective factors which may determine the beneficial role of vitamin D in our region.

## 4. Maternal Vitamin D and the Risk of Allergic Disease Development in Children

There has been growing interest in the influence of maternal vitamin D intake during pregnancy on the development of allergic diseases in children. As the insufficiency of this vitamin is high in pregnant woman several studies tried to examine the associations between a mother's vitamin D intake and the allergy risk in her child. Camargo Jr. et al. conducted a birth cohort study and in early 2006 they published results of their 2- and 3-year follow-ups. The authors reported that higher maternal intake of vitamin D was associated with a lower incidence of wheeze in the child. For each 2.5 *μ*g/day (100 IU) incremental increase in vitamin D intake the authors found a 10% decrease in risk of wheeze [8, 37]. A similar inverse association was also reported by other cohort studies [38, 39]. In addition Erkkola et al. found similar association between maternal intake of vitamin D and risk of developing asthma and allergic rhinitis in 5-year- old children [10]. All of these studies did not measure vitamin D directly but looked at maternal vitamin D intake, mostly from supplements. During pregnancy the fetus is exposed to vitamin D through cord blood supply and the ability of 25(OH)D to cross the placenta. In a recent study Camargo Jr. et al. measured cord blood 25(OH)D and found an inverse association with the risk of respiratory infections and childhood wheezing, but not with incident asthma [11].

Vitamin D deficiency is very common in pregnant women globally, but until now very little information is available on the impact of this deficiency on neonatal immune function and future risk of allergic disease [40]. The prevalence of vitamin D deficiency among pregnant women was found to be 21.2% in UK, 44.6% in Belgium, and 83.6% in China [40–42]. On a molecular level, maternal vitamin D intake during pregnancy increases the mRNA levels of the immunoglobulin-like transcripts ILT3 and ILT4 in umbilical cord blood. As these receptors are critical for the generation of T suppressor cells, this finding may point towards an early induction of immunological tolerance by maternal vitamin D intake in the developing child [43]. Future studies with longitudinal cohorts are needed to light on the vitamin D hypothesis in fetal life. A randomized trial supported by the US NIH has already started on vitamin D supplementation in pregnant women (4000 IU/day) and onset of asthma in their children; the results will be available by June 2014 [15]. Still, high vitamin D intake during pregnancy might also be harmful with respect to allergic disease development: children whose mothers had a 25(OH)D concentration during pregnancy greater than 75 nmol/L had an increased risk of atopic eczema on examination at 9 months and asthma at the age of 9 years compared with children whose mothers had a concentration of <30 nmol/L [40].

Future research should differentiate oral intake from endogenous contributions to 25(OH)D status in order to explain the immunological effects of each.

## 5. Vitamin D and Food Allergy in Children

While the incidence of asthma appears to have reached a plateau in some developed nations, many of these regions are now facing a “second wave” of the allergy epidemic, which, according to Jones et al., appears to be the rising incidence of food allergy [44]. Based on epidemiological data, the recent increase in food allergen sensitization parallels the epidemic of vitamin D deficiency caused by several factors. Factors like obesity and race, which are risk factors for vitamin D deficiency, are associated with food allergy. Although the precise biological mechanism for these epidemiologic associations is not yet known, there are hypotheses that this hormonal deficiency contributes to food allergy risk [12]. Several studies have described higher rates of food allergy among children born in seasons of low UV-B intensity (autumn/winter), associated with lower vitamin D levels [45, 46]. Other authors reported a negative association of maternal vitamin D intake during pregnancy with the risk of food allergen sensitization in early childhood [47]. Accordingly, vitamin D deficiency might contribute to early-life sensitization by further compromising the immaturity of the infant immune system.

Vassallo and Camargo Jr. proposed a “multiple-hit” model in which vitamin D deficiency, in addition to compromising immune tolerance, increases susceptibility to infections and alters microbial ecology at the gastrointestinal tract, contributing to abnormal intestinal barrier permeability. These factors might synergistically promote maladaptive allergic responses to food antigens, which manifest as food allergy in genetically susceptible subjects. The authors suggest that correction of vitamin D deficiency during pregnancy and childhood might promote immunologic tolerance, suppress proallergic immune responses, improve mucosal defenses, optimize microbial flora, and thereby limit food allergy epidemic in children [12].

## 6. Sources of Vitamin D

As mentioned previously humans acquire most of their vitamin D through sun exposure and about 10% via ingested food. Vitamin D is absorbed through the gut as either vitamin D2 (ergocalciferol) or vitamin D3 (cholecalciferol) [3].

Natural sources of vitamin D include a few foods such as fatty fish (e.g., salmon, eel, and sardine), fish liver, or cod liver oil. Some fungi such as mushrooms are a natural source of vitamin D2. Animal foods such as fatty fish, liver, fish liver oils, cheese, and egg yolks contain vitamin D3. In some industrial countries, other sources of vitamin D are fortified foods (most often milk, margarine and/or butter, and breakfast cereals) and dietary supplements.

According to European Food Safety Authority (EFSA), mean intake of vitamin D in European countries varies according to sex, age, and supplementation habits. In adults, mean intake of vitamin D from foods varied from 1.1 *μ*g/day in Spain to 8.2 *μ*g/day in Finland. The range of vitamin D intake reported from 14 European countries is considerable. In high consumers (95th percentile), intake from foods is up to 16 *μ*g/day and about 1.5-fold this value in those that consume supplements in addition to foods. For infants, mean intake from foods and supplements was available from Finland (8.9 *μ*g/day) and The Netherlands (12.5 *μ*g/day). In children 1–5 years, mean intake from foods varied from 1.7 *μ*g/day, in Denmark, to 5.6 *μ*g/day, in Greece. In older children, mean or median intake from foods only varied from 1.4 *μ*g/day in Spain and Ireland to 2.7 *μ*g/day in The Netherlands [48].

The main factors which determine 25(OH)D serum levels are skin pigmentation, sun exposure, age, gender, latitude of residence, winter season, dietary habits, and dietary vitamin D fortification [49]. Below latitude of approximately 35° North, UV-B radiation is sufficient for vitamin D3 synthesis all year round. At higher latitudes, there is no cutaneous vitamin D3 synthesis during the winter months [48].

## 7. Recommendations 

Many countries recommend the intake of supplements with vitamin D usually containing 5–25 *μ*g (200–1000 IU) cholecalciferol or ergocalciferol. The two forms differ by their side chains on the sterol skeleton [50]. Cholecalciferol is more effective than ergocalciferol in elevating total 25(OH)D concentrations and maintaining those levels for a longer time [51]. Currently, EFSA proposed a daily intake of 100 *μ*g (equal to 400 IU) vitamin D for adults including pregnant and lactating women. Vitamin D intake in children and adolescents was adapted to 100 *μ*g/day for ages of 11–17 years, considering the phases of rapid bone formation and growth in this age group. For children aged 1–10 years, the upper limit dose of 50 *μ*g/day was suggested, taking into account their smaller body size. For infants, the upper limit dose of 25 *μ*g/day was recommended [48].

In the UK, elderly people (>65 years) are recommended to take 10 *μ*g (400 IU) of vitamin D as a supplemental dose. The Nordic dietary vitamin D recommendation for children of 3 years to adults of 60 years is 7.5 *μ*g/day (300 IU) vitamin D. Infants younger than 3 years and adults over 65 years old are recommended to take 10 *μ*g/day (400 IU) [52].

The Institute of Medicine's Committee (IOM) in the new 2011 report on dietary requirements for vitamin D concludes that dietary reference intake for this nutrient can only be established according to bone health outcomes. The report estimated that children over 1-year old need at least 600 IU of vitamin D a day, with a maximum upper limit of 2500 IU for children aged 1–3 years, 3000 IU for children from 4- to 8-year old, and 4000 IU/day for children aged 9 or more years old. According to extraskeletal outcomes (e.g., respiratory health) the evidence is still insufficient [4].

Clinical trial results are needed to make definitive recommendations about the optimal dose of vitamin D for immune system functioning, for asthma prevention, and for the use of vitamin D with inhaled corticosteroids to prevent steroid resistance. Several trials are under way for asthma prevention (clinicaltrials.gov, identifiers NCT00920621 and NCT00856947), for steroid efficacy (NCT01248065), or for prevention of exacerbations (NCT00978315).

## 8. Conclusions

In recent years, many studies have been published on the effects of vitamin D and its role in various diseases. Furthermore, several studies have sought to determine the effect that vitamin D has on the immune system and specifically allergic diseases. It is worth noting that different in vivo and in vitro human studies have shown effects of vitamin D on allergy, asthma, lung function, airway responsiveness, and bronchodilator response. It seems that this hormone might lead to an innovative treatment of these increasingly common conditions.

An essential issue for any vitamin D intervention concerns its dosage. The currently recommended levels of circulating serum 25(OH)D are thought to be the minimum needed for bone health, but the optimal levels for immune system function, prevention of atopy, and defense against respiratory infections are still not known. It seems that maternal supplementation during pregnancy may prevent asthma and allergy and vitamin D supplementation after birth will also probably be necessary to maintain normal immune function in the long term.

The remarkable variation in prevalence of allergic diseases and asthma between European countries suggests that geographical latitude and Mediterranean diet are important factors which determine the beneficial role of vitamin D in our region. It would be advisable to measure the vitamin D serum level in children and adults who are part of high risk groups for vitamin D deficiency. Another group would be children with respiratory viral infections and atopy in early life which are at high risk for asthma in later childhood [53]. Vitamin D supplementation is only recommended for patients who have serum level less than 20 ng/mL. Randomized clinical trials regarding treatment with vitamin D supplementation will help determine the effects on the immune system and any potential role in preventing allergic disease.

## References

[1] Holick M. F. (2007). Vitamin D deficiency. *The New England Journal of Medicine*.

[2] Hewison M. (2011). Vitamin D and innate and adaptive immunity. *Vitamins and Hormones*.

[3] Lehmann B., Meurer M. (2010). Vitamin D metabolism. *Dermatologic Therapy*.

[4] Ross A. C., Manson J. E., Abrams S. A. (2011). The 2011 report on dietary reference intakes for calcium and vitamin D from the Institute of Medicine: what clinicians need to know. *Journal of Clinical Endocrinology and Metabolism*.

[5] Wjst M., Dold S. (1999). Genes, factor X, and allergens: what causes allergic diseases?. *Allergy*.

[6] Hyppönen E., Sovio U., Wjst M. (2004). Infant vitamin D supplementation and allergic conditions in adulthood: Northern Finland birth cohort 1966. *Annals of the New York Academy of Sciences*.

[7] Asher M. I., Montefort S., Björkstén B. (2006). Worldwide time trends in the prevalence of symptoms of asthma, allergic rhinoconjunctivitis, and eczema in childhood: ISAAC Phases One and Three repeat multicountry cross-sectional surveys. *The Lancet*.

[8] Camargo C. A., Rifas-Shiman S. L., Litonjua A. A. (2007). Maternal intake of vitamin D during pregnancy and risk of recurrent wheeze in children at 3 y of age. *The American Journal of Clinical Nutrition*.

[9] Holick M. F., Chen T. C. (2008). Vitamin D deficiency: a worldwide problem with health consequences. *The American Journal of Clinical Nutrition*.

[10] Erkkola M., Kaila M., Nwaru B. I. (2009). Maternal vitamin D intake during pregnancy is inversely associated with asthma and allergic rhinitis in 5-year-old children. *Clinical and Experimental Allergy*.

[11] Camargo C. A., Ingham T., Wickens K. (2011). Cord-blood 25-hydroxyvitamin D levels and risk of respiratory infection, wheezing, and asthma. *Pediatrics*.

[12] Vassallo M. F., Camargo C. A. (2010). Potential mechanisms for the hypothesized link between sunshine, vitamin D, and food allergy in children. *Journal of Allergy and Clinical Immunology*.

[13] Ginde A. A., Liu M. C., Camargo C. A. (2009). Demographic differences and trends of Vitamin D insufficiency in the US population, 1988–2004. *Archives of Internal Medicine*.

[14] Wortsman J., Matsuoka L. Y., Chen T. C., Lu Z., Holick M. F. (2000). Decreased bioavailability of vitamin D in obesity. *The American Journal of Clinical Nutrition*.

[15] Bozzetto S., Carraro S., Giordano G., Boner A., Baraldi E. (2012). Asthma, allergy and respiratory infections: The vitamin D hypothesis. *Allergy*.

[16] Baeke F., Takiishi T., Korf H., Gysemans C., Mathieu C. (2010). Vitamin D: modulator of the immune system. *Current Opinion in Pharmacology*.

[17] Sadeghi K., Wessner B., Laggner U. (2006). Vitamin D3 down-regulates monocyte TLR expression and triggers hyporesponsiveness to pathogen-associated molecular patterns. *European Journal of Immunology*.

[18] Adams J. S., Hewison M. (2008). Unexpected actions of vitamin D: new perspectives on the regulation of innate and adaptive immunity. *Nature Clinical Practice: Endocrinology & Metabolism*.

[19] Khoo A. L., Chai L. Y. A., Koenen H. J. P. M. (2011). Regulation of cytokine responses by seasonality of vitamin D status in healthy individuals. *Clinical and Experimental Immunology*.

[20] Chambers E. S., Hawrylowicz C. M. (2011). The impact of vitamin D on regulatory T cells. *Current Allergy and Asthma Reports*.

[21] Hollams E. M., Hart P. H., Holt B. J. (2011). Vitamin D and atopy and asthma phenotypes in children: a longitudinal cohort study. *The European Respiratory Journal*.

[22] Majak P., Olszowiec-Chlebna M., Smejda K., Stelmach I. (2011). Vitamin D supplementation in children may prevent asthma exacerbation triggered by acute respiratory infection. *Journal of Allergy and Clinical Immunology*.

[23] Brehm J. M., Celedón J. C., Soto-Quiros M. E. (2009). Serum vitamin D levels and markers of severity of childhood asthma in Costa Rica. *The American Journal of Respiratory and Critical Care Medicine*.

[24] Brehm J. M., Schuemann B., Fuhlbrigge A. L. (2010). Serum vitamin D levels and severe asthma exacerbations in the childhood asthma management program study. *Journal of Allergy and Clinical Immunology*.

[25] Searing D. A., Zhang Y., Murphy J. R., Hauk P. J., Goleva E., Leung D. Y. M. (2010). Decreased serum vitamin D levels in children with asthma are associated with increased corticosteroid use. *The Journal of Allergy and Clinical Immunology*.

[26] Sutherland E. R., Goleva E., Jackson L. P., Stevens A. D., Leung D. Y. M. (2010). Vitamin D levels, lung function, and steroid response in adult asthma. *The American Journal of Respiratory and Critical Care Medicine*.

[27] Xystrakis E., Kusumakar S., Boswell S. (2006). Reversing the defective induction of IL-10-secreting regulatory T cells in glucocorticoid-resistant asthma patients. *The Journal of Clinical Investigation*.

[28] Black P. N., Scragg R. (2005). Relationship between serum 25-hydroxyvitamin D and pulmonary function in the third national health and nutrition examination survey. *Chest*.

[29] Bossé Y., Lemire M., Poon A. H. (2009). Asthma and genes encoding components of the vitamin D pathway. *Respiratory Research*.

[30] Damera G., Fogle H., Goncharova E. A., Zhao H., Krymskaya V. P., Panettieri R. A. Vitamin D attenuates growth factor-induced human airway smooth muscle cell proliferation.

[31] Bossé Y., Maghni K., Hudson T. J. (2007). 1α,25-dihydroxy-vitamin D3 stimulation of bronchial smooth muscle cells induces autocrine, contractility, and remodeling processes. *Physiological Genomics*.

[32] Clifford R. L., Knox A. J. (2009). Vitamin D—a new treatment for airway remodelling in asthma?. *British Journal of Pharmacology*.

[33] Hyppönen E., Berry D. J., Wjst M., Power C. (2009). Serum 25-hydroxyvitamin D and IgE—a significant but nonlinear relationship. *Allergy*.

[34] Pinto J. M., Schneider J., Perez R., DeTineo M., Baroody F. M., Naclerio R. M. (2008). Serum 25-hydroxyvitamin D levels are lower in urban African American subjects with chronic rhinosinusitis. *The Journal of Allergy and Clinical Immunology*.

[35] Devereux G., Wilson A., Avenell A., McNeill G., Fraser W. D. (2010). A case-control study of vitamin D status and asthma in adults. *Allergy*.

[36] Arshi S., Ghalehbaghi B., Kamrava S. K., Aminlou M. (2012). Vitamin D serum levels in allergic rhinitis: any difference from normal population. *Asia Pacific Allergy*.

[37] Camargo C. A., Rifas-Shiman S. L., Litonjua A. A. (2006). Prospective study of maternal intake of vitamin D during pregnancy and risk of wheezing illness in children at age 2 years. *The Journal of Allergy and Clinical Immunology*.

[38] Miyake Y., Sasaki S., Tanaka K., Hirota Y. (2010). Dairy food, calcium and vitamin D intake in pregnancy, and wheeze and eczema in infants. *European Respiratory Journal*.

[39] Devereux G., Litonjua A. A., Turner S. W. (2007). Maternal vitamin D intake during pregnancy and early childhood wheezing. *The American Journal of Clinical Nutrition*.

[40] Gale C. R., Robinson S. M., Harvey N. C. (2008). Maternal vitamin D status during pregnancy and child outcomes. *The European Journal of Clinical Nutrition*.

[41] Vandevijvere S., Amsalkhir S., van Oyen H., Moreno-Reyes R. (2012). High prevalence of vitamin D deficiency in pregnant women: a national cross-sectional survey. *PLoS ONE*.

[42] Xiang F., Jiang J., Li H. (2013). High prevalence of vitamin D insufficiency in pregnant women working indoors and residing in Guiyang, China. *Journal of Endocrinological Investigation*.

[43] Rochat M. K., Ege M. J., Plabst D. (2010). Maternal vitamin D intake during pregnancy increases gene expression of ILT3 and ILT4 in cord blood. *Clinical and Experimental Allergy*.

[44] Jones A. P., Tulic M. K., Rueter K., Prescott S. L. (2012). Vitamin D and allergic disease: sunlight at the end of the tunnel?. *Nutrients*.

[45] Mullins R. J., Clark S., Katelaris C., Smith V., Solley G., Camargo C. A. (2011). Season of birth and childhood food allergy in Australia. *Pediatric Allergy and Immunology*.

[46] Vassallo M. F., Banerji A., Rudders S. A., Clark S., Mullins R. J., Camargo C. A. (2010). Season of birth and food allergy in children. *Annals of Allergy, Asthma and Immunology*.

[47] Nwaru B. I., Ahonen S., Kaila M. (2010). Maternal diet during pregnancy and allergic sensitization in the offspring by 5 yrs of age: a prospective cohort study. *Pediatric Allergy and Immunology*.

[48] EFSA Panel on Dietetic Products (2012). Scientific opinion on the tolerable upper intake level of vitamin D1. *EFSA Journal*.

[49] Mithal A., Wahl D. A., Bonjour J. P. (2009). Global vitamin D status and determinants of hypovitaminosis D. *Osteoporosis International*.

[50] Holick M. F., Biancuzzo R. M., Chen T. C. (2008). Vitamin D2 is as effective as vitamin D3 in maintaining circulating concentrations of 25-hydroxyvitamin D. *Journal of Clinical Endocrinology and Metabolism*.

[51] Lehmann U., Hirche F., Stangl G. I., Hinz K., Westphal S., Dierkes J. (2013). Bioavailability of vitamin D(2) and D(3) in healthy volunteers, a randomized placebo-controlled trial. *The Journal of Clinical Endocrinology and Metabolism*.

[52] Lanham-New S. A., Buttriss J. L., Miles L. M. (2011). Proceedings of the rank forum on vitamin D. *The British Journal of Nutrition*.

[53] Kusel M. M. H., Kebadze T., Johnston S. L., Holt P. G., Sly P. D. (2012). Febrile respiratory illnesses in infancy and atopy are risk factors for persistent asthma and wheeze. *European Respiratory Journal*.

